# EEG hyperscanning in motor rehabilitation: a position paper

**DOI:** 10.1186/s12984-021-00892-6

**Published:** 2021-06-10

**Authors:** Matthew R. Short, Julio C. Hernandez-Pavon, Alyssa Jones, Jose L. Pons

**Affiliations:** 1 Legs + Walking Lab, Shirley Ryan AbilityLab, Floor 24, 355 E Erie St, Chicago, IL 60611 USA; 2 Department of Biomedical Engineering, McCormick School of Engineering, Northwestern University, Chicago, IL USA; 3 Department of Physical Medicine and Rehabilitation, Feinberg School of Medicine, Northwestern University, Chicago, IL USA

**Keywords:** Hyperscanning, Electroencephalography, Rehabilitation, Motor control, Group therapy, Brain connectivity, Stroke, Traumatic brain injury, Spinal cord injury, Parkinson’s disease

## Abstract

Studying the human brain during interpersonal interaction allows us to answer many questions related to motor control and cognition. For instance, what happens in the brain when two people walking side by side begin to change their gait and match cadences? Adapted from the neuroimaging techniques used in single-brain measurements, hyperscanning (HS) is a technique used to measure brain activity from two or more individuals simultaneously. Thus far, HS has primarily focused on healthy participants during social interactions in order to characterize inter-brain dynamics. Here, we advocate for expanding the use of this electroencephalography hyperscanning (EEG-HS) technique to rehabilitation paradigms in individuals with neurological diagnoses, namely stroke, spinal cord injury (SCI), Parkinson’s disease (PD), and traumatic brain injury (TBI). We claim that EEG-HS in patient populations with impaired motor function is particularly relevant and could provide additional insight on neural dynamics, optimizing rehabilitation strategies for each individual patient. In addition, we discuss future technologies related to EEG-HS that could be developed for use in the clinic as well as technical limitations to be considered in these proposed settings.

## Introduction

Studying the human brain in social settings has revealed task-specific activation of various brain regions involved in cognition as discussed in multiple review papers [1–3]. Furthermore, functional and structural connectivity analyses have allowed researchers to examine relationships across these activated regions, providing insight on how an individual may process and interpret information. These findings have led to numerous theories on the characterization of neural systems, namely the mentalizing system (MS) and mirror neuron system (MNS) [4–6]. The MS, which primarily involves the temporal-parietal junction (TPJ) and medial prefrontal cortex (mPFC), plays a role in the anticipation of others intentions [4]. In order to code the neural representations of these intentions, the mPFC regulates and plans higher cognitive function while the TPJ provides context to a given situation. The MNS, on the other hand, is activated when preparing one’s own actions and imitating the actions of others and has been associated with the left inferior frontal and premotor cortices as well as the inferior parietal lobe [7, 8].

Evidence of these neural systems has been further explored in the context of inter-brain dynamics while recording from multiple subjects [9]. Hyperscanning (HS) is a technique that allows one to record the brain activity of two or more subjects simultaneously [10]. The first effort to record the activity of two brains simultaneously with electroencephalography (EEG) was performed by Duane and Behrendt [11]. However, the technique started to gain importance two decades ago [12]. Several HS studies have been carried out in healthy participants to understand changes in brain activity due to social interactions [12–15], including motor tasks [16, 17], speech [18], and musical performance [19–21].

However, most of the published studies have been limited to describing interactions between individuals performing simple tasks or under simple stimuli restricting the use of the technique beyond the laboratory. Therefore, to reach a deeper comprehension of the mechanisms involved in social interactions during “normal” life situations with peers it is necessary to generate experimental paradigms that are as “natural” as possible. As noted in a review by Hari and Kujala [1]; “much of the fleeting, moment-to-moment information of social interaction remains beyond the reach of studies involving limited stimuli and tasks. The current challenge for brain imaging is to bring every day human interaction, occurring in a complex natural environment between two or more subjects, into the laboratory”.

With a similar interest in studying interpersonal interactions, group dynamics have also been explored in the context of motor rehabilitation. Group therapy, defined as two or more individuals participating in specialized activities mediated by clinicians, has been used as a supplement to traditional therapy in rehabilitation settings [22, 23]. This approach to treatment provides greater peer support, resulting in improvements such as increased physical function, engagement, and quality of life in patients with various neurologic diagnoses [24–27]. Notably, group therapy settings share many parallels with the HS contexts that have been studied in dyads or groups of healthy individuals.

Consequently, HS studies have not been explored in patient populations due to the complexity of the clinical environment and the different technical challenges that need to be addressed. Thus, how motor recovery during social interactions in patients is reflected through changes in brain connectivity, for instance in a group therapy setting, has yet to be investigated. In this paper, we propose an approach to study EEG-HS in different patient populations, such as stroke, spinal cord injury (SCI), Parkinson’s disease (PD), and traumatic brain injury (TBI). In addition, we address different combinations of dyads during motor rehabilitation such as Patient–Patient, Patient–Therapist, Patient–Healthy and Patient–Machine. Here we focus only on EEG-HS because of its high temporal resolution, affordability and high mobility in comparison to other neuroimaging techniques such as functional near-infrared spectroscopy (fNIRS), functional magnetic resonance imaging (fMRI), or magnetoencephalography (MEG).

## Hyperscanning modalities

Many early neuroimaging studies focused on recording brain activity from a single participant while perceiving stimuli in order to assess cognition in a controlled, laboratory environment [5, 9]. While these studies were foundational in validating neuroimaging technologies and answering many questions related to sensory function, they failed to address the two-person neuroscience described by Hari and Kujala [1]. This follows the idea that, during interpersonal interaction between two or more individuals, time-varying relationships in brain activation may arise and reveal important findings regarding inter-brain dynamics. Because daily-life activities are most often experienced in dyads or groups of people, studying simultaneous activity from multiple brains has particular relevance in providing a more complete understanding of social cognition. In order to capture these phenomena, neuroimaging techniques traditionally used in single-subject recordings were configured for multi-subject applications [9, 28–30].

HS has been validated with four primary functional neuroimaging techniques: fMRI, fNIRS, EEG, and MEG [31]. Each of these methods are more or less suited for measuring brain activity during specific tasks due to their respective recording principles. fMRI offers the highest spatial resolution of the four techniques, localizing hemodynamic activity from the cortex to deeper brain structures [32]. However, due to the size constraints of the scanner as well as its comparatively low temporal resolution, fMRI-HS paradigms are limited to studying small, isolated behaviors and decision-making tasks. MEG measures magnetic fields produced by electrical potentials in the brain, providing high spatial and temporal resolution [33]. However, similar to fMRI, applications in HS research are limited due to the movement constraints that these devices impose. In addition, both fMRI and MEG systems are very expensive and may require large, dedicated spaces for installation and operation.

The two modalities most suitable for studying movement-related paradigms during social interactions are fNIRS and EEG [30, 34]. These modalities have been integrated into wearable systems allowing for virtually unrestricted movement from the participant. fNIRS is less susceptible to noise as it is an indirect measure of hemodynamic activity in the brain. On the other hand, developments in artifact detection have greatly improved the quality of the signals obtained with EEG. Therefore, while fNIRS and EEG both measure signals from the cortex with comparable spatial resolution, EEG has a higher temporal resolution (sub-milliseconds), allowing for analysis of certain behaviors on a smaller timescale [35, 36]. For example, fNIRS could be used to measure changes in cortical activity during walking compared to rest while EEG is capable of measuring intra-stride changes in cortical activity during walking. This difference is especially important when considering the degree of resolution needed to quantify planning, coordination and movement-execution involved in interacting dyads.

## EEG hyperscanning

### Data acquisition

EEG has become an indispensable tool for basic brain studies and clinical applications, and the instrumentation is much less expensive than that of other techniques, such as MEG and fMRI [37]. In addition, the high temporal resolution of EEG and its mobile capabilities have many advantages in comparison to other imaging techniques, for instance, it is simpler to record two or more subjects simultaneously with EEG-HS [9, 34]. With these advantages in mind, we will focus our discussion on EEG as we believe it is the most appropriate neuroimaging modality for the applications discussed later in this paper.

EEG-HS data have been recorded with 28–64 EEG channel systems (10–10 or 10–20 montage and active or passive electrodes) at a sampling frequency ranging from 200 to 5000 Hz [13, 15, 38–40]. From a recording standpoint, many commercial EEG systems can accommodate HS paradigms. Well-designed EEG-HS experiments require precise time synchronization across all recording equipment (e.g., presentation software, motion capture systems, video recordings). In many cases, a defined set of external stimuli or markers is used to correlate observed behaviors with the measured brain signals. Generally, this is accomplished one of two ways when recording from two or more participants simultaneously: (1) two recording computers with two separate EEG amplifiers, connected to a master computer and synchronized via an external trigger or (2) one recording computer and amplifier with electrode bundles split between two participants. In the first case, the precise synchronization between the two recording computers is crucial as delays between the two EEG recordings could potentially lead to inaccurate interpretations of the data. A recent review paper [29] provides more detail regarding specific EEG systems (i.e., Brain Products, ANT, EGI, and BioSemi) and how they have been used in HS studies. Overall, data acquisition will follow the same procedure as typical, single-subject EEG recordings with the additional consideration of time synchronization between EEG devices if using separate systems.

### Data analysis

The analysis of HS data is challenging and involves many technical considerations (e.g., removing artifacts, choosing an unbiased estimator, defining rigorous experimental controls) that should be addressed to perform an accurate interpretation. The most straightforward approach is to use the intra-brain data analysis techniques (i.e., those used for analyzing data from single subjects) and adapt them to analyze inter-brain HS data (i.e., data recorded from 2 or more brains). Nevertheless, it is difficult to separate inter-brain relations related to identical stimuli presented to both participants from relations that represent between-brain networks [31, 41]. When applying the HS technique to the motor rehabilitation paradigms discussed in later sections, we advocate for first performing offline analysis of HS data. Once substantial evidence of inter-brain measures has been established for a given HS paradigm, an online analysis may be used to develop tools for real-time evaluation of interpersonal dynamics between interacting individuals.

HS studies typically report inter-brain synchronization (or brain-to-brain synchrony) in order to describe significant causality and correlation in brain activity between dyads or groups of participants interacting. The measures that quantify this phenomenon involve comparisons between signals in the time and/or frequency domain [31]. The most widely used methods to assess EEG-HS connectivity include the following: (1) Phase coherence, (2) Phase locking value (PLV), (3) Phase lag index (PLI), (4) Granger-causality, (5) Partial directed coherence (PDC), and (6) correlation [31].

For example, correlation between signal amplitude or power in various frequency bands has been used to characterize synchrony [42]. Common coupling estimators like PLV [17] and phase coherence [43] are used to measure the phase differences in signals, though the potential bias of these measures has been discussed [41]. Mutual information, the joint dependence between two or more variables, has been used to characterize the causal links between participants in the time-domain with Granger-causality [44] and Kraskov mutual information [41] and in the frequency domain with PDC [12, 15]. In well-designed experiments, all measures are computed and compared across multiple conditions in order to control for similarities that may be coincidental in nature or related to the observation of similar stimuli. This experimental consideration is demonstrated in an EEG-HS study of finger tapping in dyads, where dynamic coordination between human participants was compared to coordination between each participant and a computerized metronome [39]. In both conditions, participants were seated back-to-back and only received auditory feedback of tapping sounds, ensuring that the same external stimuli were preserved between conditions.

Classification analyses with supervised learning can be performed as well in order to characterize the accuracy of different connectivity measures used for discrimination between behavioral conditions. For instance, Support Vector Machines have been used in a previous HS study to characterize the contributions of inter-brain and intra-brain connectivity measures in classifying dyadic interactions compared to individual interactions with computers during a visuomotor task [45]. Another study used a classifier based on Riemannian geometry to discriminate between different emotional states of interacting partners [46].

## Hyperscanning in social interactions

Over the past two decades, studies in the field of neuroscience have explored how multiple individuals interacting with one another results in inter-brain synchronization. As a result, numerous review papers have been recently published describing the various social contexts that have been studied using HS setups [14, 31, 47–51]. The review published by Wang et al. groups these social contexts into six primary domains: imitation, coordination, eye contact, game theory, cooperation or competition, and natural scenarios.

Imitation involves tasks where one participant is instructed to mirror the observed actions of another participant. Taking this concept a step further, coordination involves joint action or synchronized behaviors between participants. In these paradigms, participants are often assigned roles of “leader” and “follower” or “model” and “imitator”. Eye contact tasks analyze the exchange and processing of non-verbal, social cues through eye-to-eye contact or mutual gaze of a common object. Game theory is a field of study that includes economic exchanges, trust-building exercises and interactive decision making. Tasks that involve cooperation or competition measure the differences between dyads that work together and against one another through turn-based games or timed behaviors. Finally, natural scenarios attempt to study social interactions as they occur in everyday life.

In addition to HS studies that focus on natural scenarios, efforts to capture more naturalistic paradigms have been considered across all domains. Most HS setups are conducted in enclosed laboratory spaces and involve many repetitions of a single task. These conditions often do not mirror the activities experienced in everyday life and therefore the results may not be directly reflective of social cognition as it “naturally” takes place. In addition, results from these repetitive experimental tasks can be affected by the engagement of the participant. Previous review papers have suggested modifying experimental setups to account for these discrepancies in order to contribute to a better understanding of social interaction through HS [14, 31].

Another important consideration in these HS studies is the effect of emotion and personal connection that spans each domain. Studies have demonstrated higher connectivity and performance in romantic partners versus strangers in button pressing tasks [44] and in creative tasks [52] as well as higher synchrony when children interact with their parents versus a stranger during a cooperative activity [53]. In addition, discrete regions of inter-brain coherence have been identified between same sex and mixed-sex dyads during a cooperative computer task [54]. These findings show the importance of not only the designed task and assigned roles on inter-brain synchronization, but also the relationship between the individuals engaged in the task. Because personal connections can influence both the degree and location of observed synchrony in the brain, these factors should be explored in additional contexts (e.g., age, gender, disability) and paradigms in order to better characterize HS results. Conversely, if these emotional connections are not the focus of a study, the experimental design should make an effort to control for these potential confounding factors related to distinct dyads whenever possible.

## Hyperscanning in movement-related paradigms

When considering the application of existing EEG-HS paradigms to motor rehabilitation in patient populations, it is important to note relevant results of movement-related paradigms in neurotypical individuals. So far, simple, upper extremity movements and gestures have been studied with HS setups which fall under the contexts of imitation and coordination as previously mentioned. Additionally, most of these studies focus on the central-parietal region of the brain when reporting inter-brain synchronization.

One EEG-HS study explored the relationship between behavioral and neural synchronization through imitation of hand gestures in dyads [17]. In this study, one participant was instructed to imitate the other participant’s hand movements under two conditions: spontaneous (self-determined) and induced imitation. For spontaneous imitation, inter-brain synchronization was estimated using PLV and revealed right centro-parietal networks in the alpha frequency band across participants during the exchange of gestures, consistent with the theories of the MNS. Interestingly, significant synchrony was also identified during periods of imitation where the gestures were not exactly mirrored in terms of hand shape and direction of movement, indicating that inter-brain synchronization may not exclusively depend on the precise execution of a particular movement. These findings suggest that self-determined engagement during gestural imitation is more informative than the ability to perfectly mimic a given gesture, as quantified by inter-brain synchronization. Future studies could explore if these findings hold during more complex gestures or movements involved in rehabilitative exercises discussed in the next sections.

Another EEG-HS study explored social coordination involved with rhythmic finger movements [16]. While pairs of participants observed one another’s self-paced finger movements in real-time, two oscillatory components in the right centro-parietal cortex were identified using a measure similar to the PLV. These components were concluded to have opposing functions as one was associated with enhancement of independent movements (inhibition of MNS) and the other was associated with enhancement of coordinated movements (excitation of MNS). The same research group expanded on these findings with a focus on mu band power modulation [42]. They found that changes in mu band power were identified during rhythmic finger movements under different contexts (e.g., anti-phase, in-phase, intrinsic), emphasizing the role of the right-central parietal region in mediating the interpretation and imitation of others’ actions. Together, the findings from these studies [16, 17, 42] also emphasize the importance and focus on the mu (alpha) band in analysis of HS measures during coordination and imitation.

**Fig. 1:**
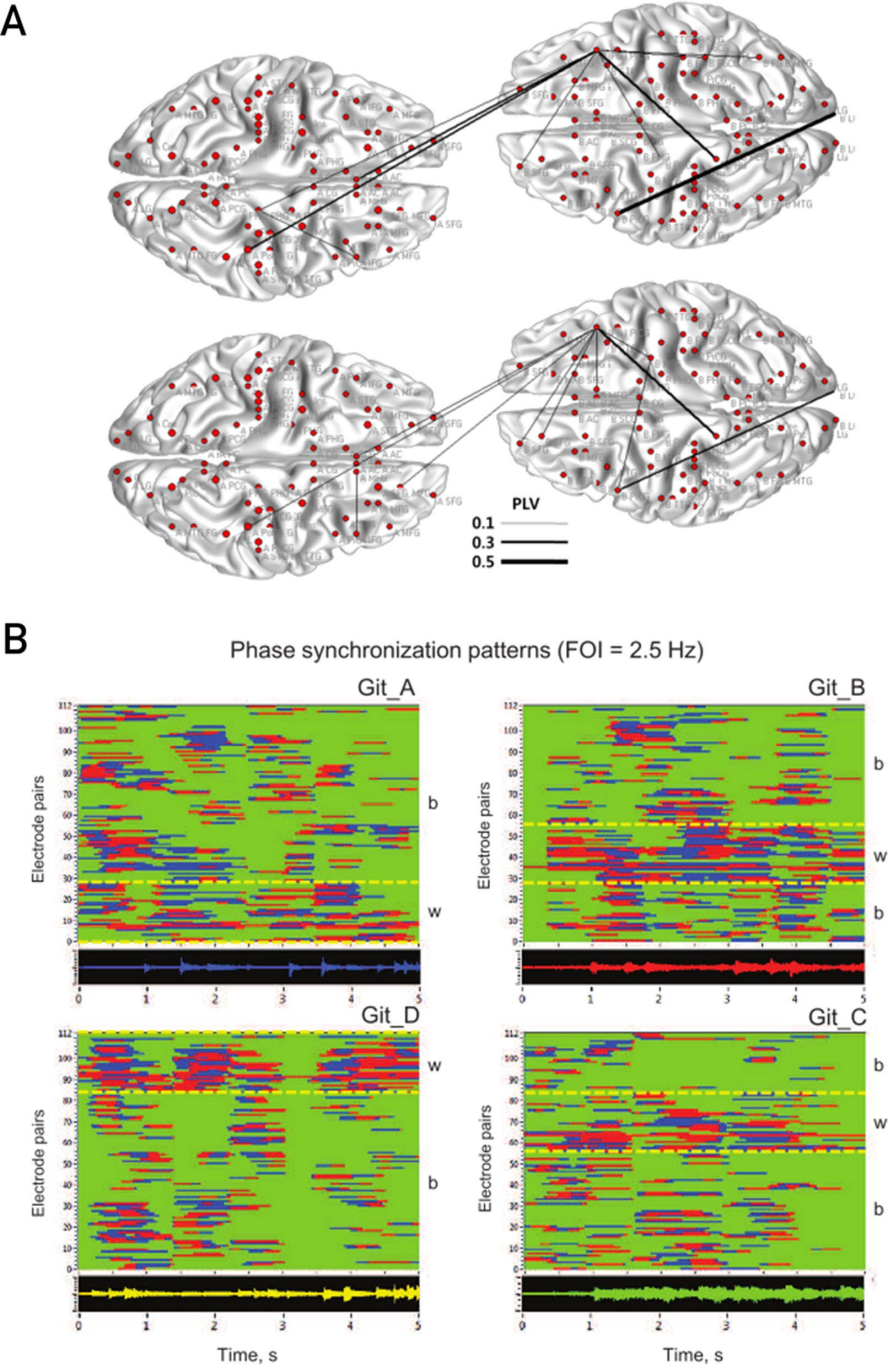
Methods of data analysis used in electroencephalography hyperscanning (EEG-HS) during movement-related activities. **A** HS analysis between brains during unconscious finger movements quantified with phase locking value (PLV) [55], **B** Periods of phase synchronization (at 2.5 Hz) used in network analysis of musical coordination in guitar quartets [19]. **A** and **B** were reproduced and adapted with permission from copyright holders under Creative Commons licensing

To assess the basis of implicit social coordination, an EEG-HS study analyzed unconscious fingertip movements between dyads before and after cooperative training exercises [55]. The results showed that, after training, the number of functionally significant connections between dyads increased in the inferior frontal gyrus, anterior cingulate, parahippocampal gyrus and postcentral gyrus as measured by phase synchrony (Fig. 1A). In the context of motor rehabilitation, these findings are particularly important as they indicate that inter-brain synchronization can be enhanced over time through exercises that focus on cooperation between dyads. As interventions for motor rehabilitation are delivered over a period of time in conjunction with a patient’s plan of care, tracking changes in HS measures as a result of different dyadic or group interactions could be a potential application of the aforementioned study [55] to patient populations.

In addition to simple joint movements, more complex motor behaviors have been studied through musical performance with HS setups. In the context of a guitar duet, one group used an EEG-HS setup to associate musical roles of leader and follower with asymmetric periods of phasing locking at the frontal and central electrodes [21]. This study also found that phase coherence was enhanced in the frontal and central regions during sections demanding higher coordination between players, emphasizing the importance of these regions in interpersonal action coordination. The same group further investigated this paradigm in a later study using a directional measure of connectivity to assess the transfer of information between leader and follower in a guitar duet [20]. This measure revealed time-lagged periods of synchronization around the onset of playing and showed asymmetries in the strength of synchronization for the frontal and parietal regions between the players. Networks of connections have also been explored during musical performance in larger groups of musicians. Using EEG-HS, synchronous brain activity was measured in guitar quartets, revealing networks of activity characterized by both inter- and intra-brain connectivity [19]. Interestingly, the structure of these networks changed over time depending on the section of music performed and at times involved electrodes shared by two, three or all four brains of the interacting participants (Fig. 1B).

## Future considerations: hyperscanning in patient populations

**Fig. 2:**
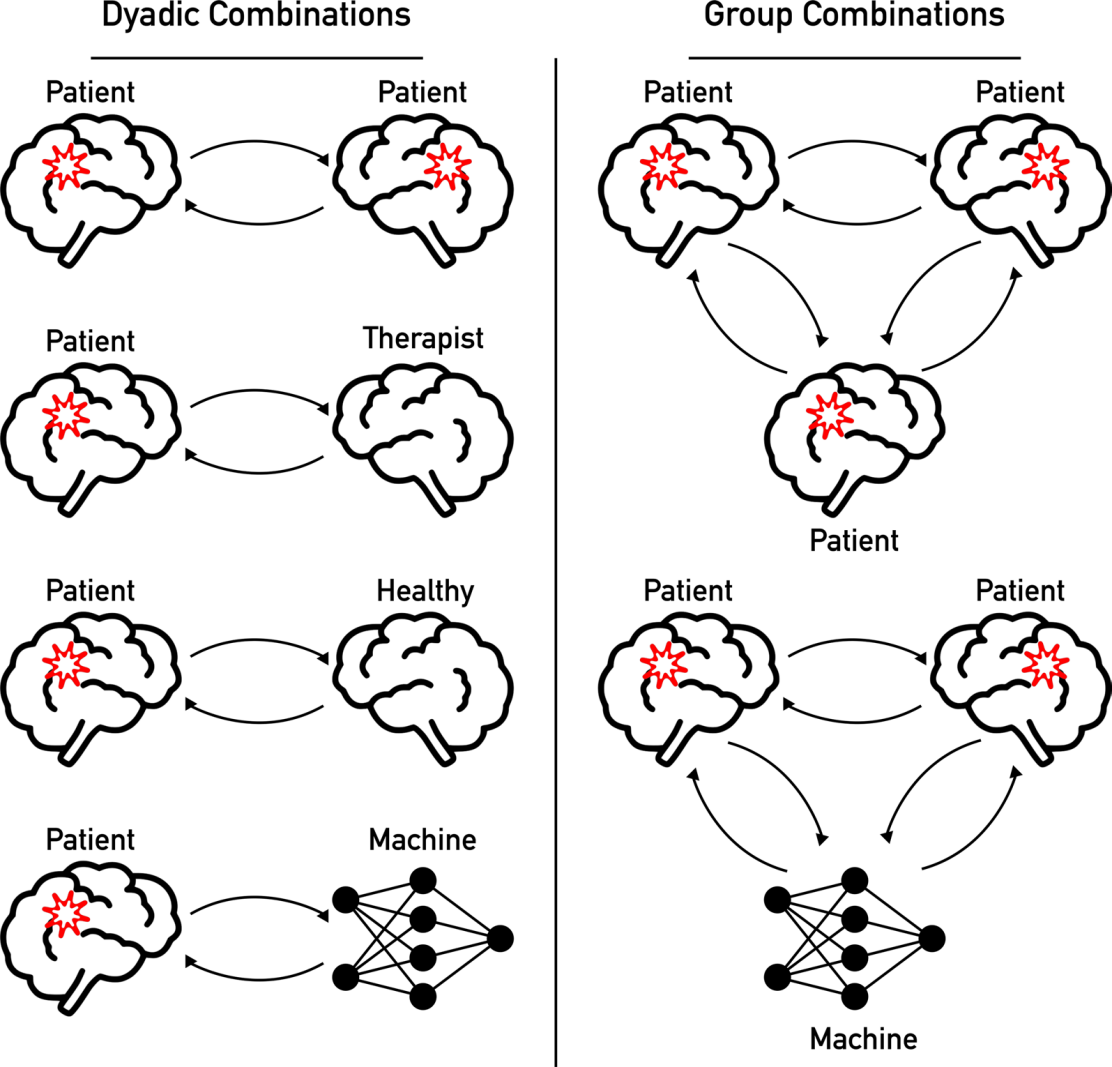
Examples of dyadic and group combinations proposed for future hyperscanning (HS) studies in motor rehabilitation settings. These dyadic combinations include Patient–Patient, Patient–Therapist, Patient–Healthy, and Patient–Machine. Group studies could be organized to include various combinations of the dyads proposed here

HS thus far has primarily focused on inter-brain dynamics in healthy participants. While the field is relatively new, it could benefit immensely from expanding to include patient populations. Comparing established results from healthy participants to similar social contexts in patient populations can be essential in determining whether or not the observed instances of inter-brain synchronization are of physiological relevance. When pursuing these comparisons, many combinations of dyads are of interest. For this paper, we have identified four potential combinations that are relevant in rehabilitation settings: (1) Patient–Patient, (2) Patient–Therapist, (3) Patient–Healthy, and (4) Patient–Machine (Fig. 2).

In Patient–Patient dyads*,* interactions between two individuals with a similar neurologic diagnosis (e.g., stroke, spinal cord injury) may be analyzed. These interactions could involve tasks that are typically performed in each patient’s treatment program (e.g., stretching exercises in a physical therapy program). Specific examples of these tasks will be discussed extensively in the following sections.

Alternatively, the same interactions could be studied in Patient–Therapist dyads to measure differences when an individual with a neurologic diagnosis interacts with a trained professional. Because the therapist has substantial expertise in the activity of interest and over time builds a rapport with their patient, these factors can potentially affect the patient’s rehabilitation outcomes as well as the interpersonal dynamics during therapy. In traditional motor rehabilitation, the therapist should consider the skill level or functional capacity of each patient and the demands of the task being performed. For patients with lower skill levels, the therapist may provide varying levels of physical assistance or cuing to help the patient successfully complete the task. As a patient begins to achieve a higher skill level, the therapist will reduce the level of assistance and progressively increase the task difficulty to elicit error augmentation and facilitate motor adaptation [56, 57]. Based on this approach, Patient–Therapist interactions could potentially involve two scenarios: mirrored movements between dyads or an interaction where the therapist guides and/or directs a patient through an exercise, depending on the patient’s functional ability.

Patient–Healthy dyads, an individual with a neurologic diagnosis paired with an untrained, healthy individual, could provide an additional comparison with the other combinations mentioned. One possibility would be to assess dyads consisting of a patient and their family member, potentially revealing advantageous, emotional connections similar to what has been explored in romantic partners [44, 52]. Comparing the interactions between a patient and their family member versus a patient and their therapist could help distinguish the effect of emotion and skill, respectively, on inter-brain dynamics and, in turn, functional improvements in rehabilitation settings.

Finally, Patient–Machine dyads, an individual with a neurologic diagnosis interacting with a computer, can be studied and compared to the baseline findings from each combination of human dyads. With development of artificial controllers (i.e., robotics) that mimic human behavior, the impact of robot-mediated therapies can be assessed in the context of these social interactions.

Establishing results on inter-brain dynamics through HS in each of these dyadic combinations can also lead to further questions and applications in larger groups. Recording simultaneous brain activity from more than two individuals has the potential to reveal networks of connections amongst patients and therapists interacting in the same clinic. If smaller groups can be identified within a larger group based on increased measures of inter-brain synchronization, this has the potential to create more effective, synergistic training groups as HS provides an additional physiological measurement of engagement. Over time, these interpersonal connections may lead to increased engagement which may be correlated with the functional outcomes of each patient such as motor learning or skill acquisition. A previous study in healthy individuals supports this hypothesis, showing that there is greater behavioral synchrony and inter-brain synchronization between basketball teammates compared to strangers during a joint drawing task [58]. Additionally, the influence of Patient–Machine interactions could be explored in larger groups as well. For instance, network analyses of hyperconnectivity could be compared in exercise groups between a group of four patients interacting together versus a group of three patients interacting with a computer that provides feedback related to the exercise. Overall, these comparisons could reveal how inter-brain connectivity is affected as a result of a neurologic diagnosis, guiding and optimizing the rehabilitation strategy for an individual patient.

## Potential application of hyperscanning: group therapy

In the following sections we will discuss the patient populations and paradigms we believe are most relevant to future HS analyses. The patient diagnoses to be considered include stroke, SCI, PD, and TBI. In each of these patient populations, some form of group therapy has been researched as an alternative or supplement to traditional, motor rehabilitation. These applications of group therapy present noteworthy opportunities to evaluate brain activity from multiple patients simultaneously and assess the functional implications.

Group therapy in rehabilitation is defined as two or more individuals participating in skilled therapy treatment with guidance from one or more clinicians [22, 23]. It is most often described in literature as one of two paradigms: (A) performance of an activity in which patients are engaged in a common, coordinated activity (e.g., partnered dance), and (B) performance of exercise or activity together in the same environment, however not necessarily in a coordinated fashion (e.g., group circuit training). Throughout the following sections, we will focus on these two forms of group therapy and refer to them as paradigms A and B.

Advantages of group rehabilitation include peer social support, increased accessibility to and opportunity for treatment, decreased staff demands, and cost-effective delivery of treatment [22, 23, 26, 59]. The elements of these groups are driven by the functional status and goals of the members, staff allocation and reimbursement from third party payers [22, 23]. Delivery of interventions in group settings can be performed by several disciplines within the interdisciplinary team, including physical therapy, occupational therapy, speech language pathology, recreation therapy, and psychology. These interventions can occur in the inpatient, outpatient, and community settings [22, 27, 60]. The delivery of interventions in the group setting has shown to be efficacious in improving physical function, cognition, mood, participation, pain, and quality of life (QOL) in patients with neurologic diagnoses including stroke [26], SCI [27], PD [24], and TBI [25].

Overall, there is a lack of neuroimaging studies in group therapy settings. Incorporating EEG-HS setups in these contexts could allow for the analysis of not only inter-brain synchronization, but also intra-brain connectivity and single-brain measurements to further characterize the effect of this specialized treatment.

### Stroke

Motor impairment is a common sequela of stroke, often resulting in the loss of functional independence and quality of life [61]. Rehabilitation focused on repetitive, specific, and intense task practice has shown to be effective in facilitating motor recovery [62]. Delivery of interventions in group settings has shown to be a cost effective way to increase intervention dosage, especially in the crucial early days of stroke rehabilitation [59, 61].

Studies have investigated interventions using both paradigms A and B [26, 59, 61, 63–65]. As an example of paradigm A, Van Vugt et al. compared the effect of dyads post-stroke playing simple piano songs synchronously versus in-turn (one after the other) on improving fine motor function in the hemiparetic upper extremity [63]. They hypothesized the group that played synchronously would be more socially engaged with their partners, resulting in improved rehabilitation outcomes. The results revealed both groups improved fine motor control and demonstrated reductions in depression and fatigue, however, the in-turn group tended to demonstrate greater motor improvement in addition to reporting greater sympathy towards their partners and positivity regarding the training sessions.

Group therapy interventions in stroke rehabilitation investigating tasks consistent with paradigm B have also been examined. Interventions include modified constraint induced movement therapy, circuit exercise training, and robotic assisted therapy, occurring both in inpatient rehabilitation and outpatient community settings [26, 59, 61, 64, 65]. These interventions resulted in significant improvements in measures of upper and lower extremity function in both subacute and chronic stroke. For instance, Hesse et al. demonstrated that robot assisted group therapy was just as effective as individual arm therapy in improving upper motor extremity function, but required decreased staff demands [59].

### Spinal cord injury

SCI involves damage to the spinal cord, often affecting motor and sensory function as well as quality of life. Currently, there is limited evidence describing the efficacy of group therapy on functional outcomes in individuals with SCI. However, Zanca et al. reported findings from the SCIRehab study analyzing group therapy data collected from over 1300 patients with traumatic spinal cord injury over five years at inpatient rehabilitation hospitals in the United States [22]. The type of group treatment (e.g., physical therapy, occupational therapy, recreation therapy, and psychology) and amount of time spent in group therapy varied among patients based on the individual levels of injury and functional impairments. Notably, individuals with incomplete SCI spent more time in physical therapy groups compared to individuals with complete SCI, focusing on range of motion/stretching, balance, wheelchair skills, education, and most commonly strength and endurance training. While no data were reported on the impact of participation in group therapy on functional outcomes, two additional studies have shown group therapy delivered in the form of a community exercise class is feasible and resulted in improvements in health, mood, pain, perceived health status, and regular participation in exercise for individuals with SCI [27, 66].

### Parkinson’s disease

PD is a progressive neurodegenerative disorder characterized by motor symptoms including bradykinesia, tremor, rigidity and postural instability and nonmotor symptoms such as cognition, mood, pain, sleep disorders, and fatigue [24]. Compared to pharmacological and surgical interventions, physical activity and exercise have been shown to be advantageous treatments that address both the motor and nonmotor symptoms of PD discussed above [24]. Furthermore, several studies have revealed that exercise and physical activity interventions administered in groups to be effective in improving physical function, participation in exercise, self-efficacy, mood, QOL, cognition, fall frequency, and disability in individuals with PD [24, 67–75].

Group therapy intervention investigated in PD has occurred in rehabilitative and community settings and can be characterized by both group task paradigms, A and B. For example, partnered dance such as Argentine Tango is an example of an intervention where individuals are engaged in a common, coordinated activity (paradigm A) and has been supported by numerous studies as an efficacious intervention in improving both motor and nonmotor symptoms related to PD [24, 67–69, 76]. It is believed that dance is an effective intervention due to its ability to challenge dynamic balance, aerobic capacity, trunk and limb range of motion, cognition, memory, and dual tasking while providing external auditory cues from music and external visual cues from partners [68, 69].

Group therapy interventions consistent with paradigm B include Tai Chi, circuit training, boxing, and PD specific exercise programs, delivered in a group class setting [71–75]. These interventions resulted in significant improvements in motor symptoms, function (i.e., balance, gait speed, endurance), mood and QOL, in addition to increasing adherence and accessibility to exercise specific to individuals with PD [70–75].

### Traumatic brain injury

TBI can result in motor and neuropsychological impairments including cognition, emotion regulation, attention, memory, reasoning, and self-awareness [25, 77]. These impairments often result in difficulty with reintegration into the community, decreased functional independence and reduced QOL. Although limited, studies specifically investigating multidisciplinary rehabilitation and exercise interventions administered in the group format in the rehabilitation and community settings have shown to be effective in improving physical function, cognitive function, and disability in individuals post TBI [25, 77–80]. With regards to addressing motor function in TBI, group therapy intervention investigating paradigm B involve Yoga, Tai Chi, and circuit training delivered in a group format in the rehabilitation and community settings [78–82]. These interventions resulted in significant improvements in motor function (i.e., balance, strength, and endurance), respiratory function, community integration, mood, self-esteem, and QOL, in addition to increasing adherence and accessibility to exercise specific to individuals with TBI.

## Open question: what are the implications of hyperscanning in motor rehabilitation?

Given the recent growth of the two fields, we believe merging HS techniques with group therapy interventions could result in considerable benefits in rehabilitation settings as well as in motor and cognitive neuroscience research. Considering the existing body of HS literature, it is clear that there are many parallels between the social contexts of imitation and coordination studied in healthy participants and the rehabilitation strategies implemented in patient populations involved in group therapy. Because many of these group therapy interventions have established protocols within the clinic, the application of HS only involves a modification to existing experimental setups. EEG recordings, for example, can be implemented in addition to the measurements that may already be collected (e.g., heart rate, blood pressure). This provides a relatively straightforward way to capture natural, interpersonal interactions as described by Hari and Kujala [1].

Instances of group therapy where patients are engaged in a common, coordinated activity (paradigm A) were discussed in the previous section for stroke and PD. For example, the experimental setup studied in Van Vugt et al. can potentially be replicated utilizing EEG to measure brain connectivity between dyads post-stroke as a supplement to the measures of engagement and sympathy that were originally reported [63]. With the piano duets described in this study, it is likely that there is some directional flow of information between brains (i.e., leader and follower roles [20, 21]) that results in increased synchronization and can be measured via EEG-HS setups. As a further analysis, inter-brain synchronization during sections of music with varied complexity can be analyzed and compared. Altogether, this information could be used to determine optimal intervention parameters (e.g., type of music, combination of partners) to maximize rehabilitation outcomes. Particularly in patients with stroke and TBI, a relevant question to be answered with HS is whether or not increased inter-brain synchronization between patients (or patients and therapists) corresponds to greater changes in functional motor improvement and neuroplasticity. With longitudinal studies, this can be tested over multiple sessions of group therapy in a patient’s rehabilitation plan of care using functional measurements of brain activity to assess long-term changes in connectivity.

In PD, EEG-HS could provide an opportunity to further investigate the mechanisms of cortical activation during partnered dance. Related to previous HS studies in healthy individuals demonstrating greater inter-brain synchronization between individuals with familial or romantic connections [44, 52, 54], individuals with PD reported greater enjoyment and sense of achievement when paired with compatible dance partners such as spouses or friends versus unknown volunteers [83]. HS would provide means to investigate the neural coupling between different types of dance partners in PD and their potential impact on nonmotor and motor functional outcomes.

Group therapy rehabilitation strategies consistent with paradigm B were previously discussed for all neurological conditions described in this paper. HS experiments can be used to evaluate the inter-brain dynamics in these contexts as well, presenting an opportunity to measure and characterize networks of interacting patients. Similar to one group HS study performed in a classroom setting [84], pairwise comparisons as well as group comparisons of synchrony could be used to evaluate engagement in the group exercise classes and as a predictor of individual, functional improvements in each of the patient populations. Because this form of group therapy is similarly applied in all of the patient population discussed, implementing HS setups could help refine the approach to exercise groups as a whole, developing comprehensive findings that span multiple neurological populations during this type of social interaction.

In addition to these specific examples of potential applications, HS in these patient populations involved in group therapy can provide a quantitative measure of engagement as a supplement to the information that is already collected. Surveys or questionnaires are used to evaluate the experience of the patient as a result of their participation in the group in many of the group therapy studies discussed [63, 66, 67] as well as in rehabilitation gaming contexts [85, 86]. This has led some groups to conclude that certain dyadic combinations are preferable over others, such as a patient and a family member or friend versus a patient and an occupational therapist during a competitive gaming task [85]. However, comparing these subjective measures of preference with measures of brain activation and inter-brain synchronization may lead to different conclusions as subjective engagement and comfort may not result in the greatest functional improvements in rehabilitation settings.

## EEG hyperscanning limitations

### Subject-specific differences

For neurological diagnoses involving brain injuries (i.e., stroke, TBI), the heterogeneity of lesions makes it more challenging to directly compare results across patients. In patients post-stroke, lesions labeled as similar regions can vary considerably in their exact location, resulting in different patterns of brain activation during motor tasks as identified by time–frequency analysis with EEG [87]. In addition, different lesion profiles have contrasting implications for functional outcomes [88] and motor excitability [89]. In TBI, subject-specific variability in lesion abnormalities has been emphasized in past studies using computed tomography (CT) imaging [90].

Because of these differences in neurophysiology across individuals within each patient population, sensor-space EEG analyses may not be appropriate when considering HS in these contexts. Due to lesions and the reorganization of the brain following injury, it is not valid to assume that the signals from one electrode placed on an individual can be directly compared to the same electrode on another participant during simultaneous recording. This issue of volume conduction has been discussed in previous papers related to modeling of brain activity in the presence of structural abnormalities [91, 92]. To combat this, we alternatively suggest source-based estimation of brain activity with techniques such as minimum-norm estimate (MNE) [33, 93], low resolution electromagnetic tomography (LORETA) [94], or any other spatial filters [95], as well as blind source separation techniques such as independent component analysis (ICA) [96]. Additionally, individual anatomies obtained via structural MRI or CT should be used in the source estimation as opposed to template images [97, 98]. In this way, subject-specific differences can be preserved in the analyses, resulting in more accurate and useful conclusions when considering comparisons between patients or participants.

### EEG artifacts: motion, muscle and noise

Recording artifact-free EEG data is almost impossible even in a well-controlled environment. Typically, the EEG recordings consist of brain signals plus a variety of non-neural noise sources. The non-neural signal consists of induced electrical noise from the recording environment (e.g., power line noise, computer monitors, mobile phones, elevators noise) and biological signals (e.g., eye movements, blinks, heart activity, muscle activity from the head and neck area, extraneous body movements). Because our proposed applications involve tasks requiring high mobility in many cases, the influence of muscle and motion artifacts on EEG signals should be considered. Additionally, working with patient populations can amplify these artifacts due to potential loss of motor control, resulting in less predictable movements and errors. These movement artifacts should be controlled for in both experimental setups as well as during post-processing of collected EEG data. Fortunately, many of those artifacts can be removed or reduced during offline data analyses. For instance, the large and transient artifacts can be eliminated by removing the bad trials that contain them. Furthermore, additional electrodes can be used to record ocular and movement artifacts. The information recorded by these electrodes can be used to separate the artifacts from the brain signals by using ICA or principal component analysis techniques [96, 99–101].

In addition, it is good practice to perform online monitoring of EEG signal quality before and during EEG experimentation. Data can be visualized through a graphical user interface, included with most commercial EEG systems, in order to detect any of the aforementioned spurious artifacts [102]. Detection of these artifacts before recording data can help identify and remove potential sources of noise in the recording environment, mitigating the need for additional artifact removal steps in offline data analysis. In the context of EEG-HS, monitoring of noise and artifacts is especially relevant. For instance, if artifacts are detected in the EEG recordings of one participant, but absent in the recordings of the partner, this could potentially influence the outcome of connectivity measures and lead to inaccurate interpretation of the interaction being studied.

### Interpretation of results

The aforementioned review papers also detail important considerations related to interpretation of results and experimental design when conducting HS analyses [14, 31]. One of the main issues is that similar phenomena in brain activity can occur independently in subjects irrespective of the interaction being studied. These phenomena could be caused by a common external stimulus or pure coincidence due to common oscillatory frequencies in the cortex across participants. Therefore, when using the hyperconnectivity measures discussed in this paper, there is a risk of describing spurious inter-brain synchronization. This issue has been demonstrated in a previous paper with simulated EEG-HS data, showing that certain measures of quantifying synchronization are subject to bias depending on the context of interaction [41]. To confidently determine whether or not changes in oscillatory activity are caused by the interaction being studied, it is essential that the experimental setup is well-controlled and compares inter-brain dynamics across multiple conditions (as previously discussed) with a measure that is insensitive to slight deviations in each individual’s oscillatory activity.

Another important detail concerning these analyses is the interpretation of significant inter-brain synchronization. As noted by Babiloni and Astolfi, instances of synchrony represent “an indirect chain of events that starts from the particular cerebral regions of the first subject and ends in the cerebral processes elicited in the brain of the second subject” [14]. HS results should therefore not be interpreted as direct communication between brains.

### Expanding to the periphery

While EEG-HS has provided useful insight on neural dynamics at the cortical level, social interactions may also involve synchronization in the periphery, especially during movement-related activities. As a means of supplementing the cortical information obtained from EEG-HS data, measures from the periphery may be compared using the same principles of HS. In their review, Hari and Kujala noted that inter-brain synchronization may also be reflected in measures from the autonomic nervous system such as cardiac and respiratory patterns [1]. In fact, it has been shown that heart rate variability and respiration are coupled across singers in a choir as measured by phase synchronization [103]. These measures would likely covary with inter-brain synchronization and could help to further characterize physiological linkages between dyads or groups of individuals.

In the context of motor rehabilitation, surface electromyography (EMG) is a common peripheral measurement used to estimate muscle activation during movement [104, 105]. EMG has been used to explore the common neural drive to groups of muscles through intra-muscular coherence [106, 107] as well as the cortical input to groups of muscles through cortico-muscular coherence [108, 109]. Given this previous work, EMG-HS measures of coherence may be adapted from single- to multi-subject analyses, similar to what was discussed in EEG-HS. While EEG-HS can provide information related to the roles of a given task and the engagement of the interacting individuals, EMG-HS could potentially supplement this information with measures related to the planning and execution of specific, coordinated movements. In addition, EEG-HS and EMG-HS can be considered as complementary measures at the cortical and cortico-spinal level, respectively.

## Conclusions

Throughout this paper, we assessed the current field of HS research and proposed opportunities to apply this technique in new contexts with patient populations such as stroke, SCI, PD, and TBI. EEG-HS, in particular, was highlighted given its utility in studying movement-related paradigms [17, 19–21, 42, 55]. While we focused on group therapy because of its growing prevalence in motor rehabilitation and close parallels to many of the social interactions previously studied in healthy individuals, the HS technique can and should be expanded to other contexts outside of the motor rehabilitation and specific populations mentioned. Though not explicitly discussed, there are many instances of psychosocial group interventions in TBI and stroke that could just as well be explored using HS setups [22, 60, 110]. Furthermore, many of the social interactions studied in healthy individuals, movement-related or otherwise, can be replicated in individuals with motor and cognitive impairments to help strengthen the findings related to inter-brain dynamics during these tasks.

In practice, HS in rehabilitative settings provides an opportunity to collect additional information from patient populations to complement commonly used physiological measurements and qualitative data. Together, these data can help clinicians make more informed decisions regarding each patient’s rehabilitation plan of care. Once well-established results are produced for HS in individuals with neurological diagnoses, new technologies can be adapted for use in the clinic. For instance, using a limited set of EEG electrodes placed in key region of interest on the scalp, brain activity can be monitored from a patient throughout a typical day or session of rehabilitation. In a clinic that incorporates some amount of group therapy, monitoring brain activity between patients has the potential to identify dyads of patients or patients and therapists that optimize both behavioral and inter-brain synchrony. With further development, technology such as this can serve as a real-time diagnostic tool for monitoring patients’ engagement and cognitive state, thus maximizing the time spent in treatment or therapy.

In addition to measurements of brain activity, the principles of HS may also be applied to other physiological measures in the periphery (e.g., muscle activity) to further characterize the neural dynamics of physical interactions. The relationship between each HS measure (e.g., EEG-HS, EMG-HS) should be studied extensively in order to fully understand the nature of these interpersonal interactions. While new measures like EMG-HS would first need to be validated in dyads or groups of healthy individuals, their implementation could be particularly relevant and useful for further characterizing and treating motor impairments in groups of patients with neurological conditions.

## Data Availability

Not applicable.

## Abbreviations

CT: Computed tomography
EEG: Electroencephalography
EEG-HS: Electroencephalography hyperscanning
EMG: Electromyography
fMRI: Functional magnetic resonance imaging
fNIRS: Function near-infrared spectroscopy
HS: Hyperscanning
ICA: Independent component analysis
LORETA: Low resolution electromagnetic tomography
MEG: Magnetoencephalography
MNE: Minimum-norm estimation
MNS: Mirror neuron system
mPFC: Medial prefrontal cortex
MS: Mentalizing system
PD: Parkinson’s disease
PDC: Partial directed coherence
PLI: Phase lag index
PLV: Phase locking value
SCI: Spinal cord injury
TBI: Traumatic brain injury
TPJ: Temporal-parietal junction
QOL: Quality of life
